# Nitrogen uptake from soil residual fertilizer by subsequent crops: regulatory effects of previous nitrogen application rate and subsequent irrigation regime

**DOI:** 10.3389/fpls.2026.1890496

**Published:** 2026-09-15

**Authors:** Xiaohui Xu, Jingnan Chen, Yiting Cai, Ying Tian, Erzi Zhang, Maomao Hou, Fenglin Zhong, Junyang Lu, Jiajia Li

**Affiliations:** 1 College of Agricultural Science and Engineering, Hohai University, Nanjing, Jiangsu, China; 2 College of Horticulture and Landscape Architecture, Fujian Agricultural Vocational and Technical College, Fuzhou, Fujian, China; 3 College of Horticulture, Fujian Agriculture and Forestry University, Fuzhou, Fujian, China; 4 Jiangning District Water Affairs Bureau of Nanjing City, Nanjing, Jiangsu, China; 5 Plant Research and Development Laboratory, S’yong Cosmetics Manufacturing Co., Ltd., Changsha, Hunan, China

**Keywords:** facility agriculture, irrigation regime, nitrogen use efficiency, pakchoi, tomato

## Abstract

**Introduction:**

Soil residual fertilizer nitrogen (SRFN) represents an important nitrogen resource that can be reused by subsequent crops.

**Methods:**

In this study, two independent ^15^N tracing experiments were conducted under two crop rotation systems (“pakchoi-pakchoi” and “pakchoi-tomato”) to investigate the effects of previous nitrogen application rates (80, 120, 160 kg N/ha) and subsequent irrigation regimes (for pakchoi: 60%, 70%, 80% of field capacity; for tomato: 50%-60%-60%, 60%-70%-70%, 70%-80%-80%) on the uptake and utilization of SRFN by subsequent crops.

**Results:**

The results showed that 2.48%–8.95% of the applied fertilizer nitrogen was absorbed by the subsequent crops. Under different previous nitrogen application rates, the maximum ^15^N uptake by subsequent pakchoi and tomato reached 3.97 kg/ha and 10.46 kg/ha, respectively. Among the irrigation regimes, the lower limits of 70% (for pakchoi) and 60%-70%-70% (for tomato) most significantly promoted root dry matter accumulation (*p* < 0.05), increased SRFN uptake by 0.45–1.01 kg/ha and 1.94–2.56 kg/ha, respectively, and achieved the highest fertilizer nitrogen reuse rates (4.05% and 8.95%, respectively). Correlation analysis further revealed that the efficiency of SRFN uptake and utilization by crops was closely related to the distribution, form, and quantity of SRFN in the soil, as well as the root biomass of the subsequent crops.

**Discussion:**

In conclusion, the previous nitrogen application rate and subsequent irrigation regime regulate the uptake and utilization of SRFN by subsequent crops through influencing the soil status of SRFN and root growth. The “pakchoi-tomato” rotation combined with the 60%-70%-70% irrigation lower limit was the most effective in promoting crop uptake and utilization of SRFN.

## 1 Introduction

Globally, protected cultivation overcomes the limitations of natural conditions and enables the cross-regional and off-season cultivation of vegetables. In China, the protected vegetable cultivation area accounts for 12% of the total crop cultivation area, and with policy guidance and capital investment, the protected cultivation area is still expanding (Li et al., 2017; Zhang et al., 2018). The synergy of air temperature and soil temperature under protected cultivation is poor. This resulted in the release of soil N nutrients lagging behind the crop demand. Thus, more N fertilizer must be applied to protected cultivation than to field cultivation to ensure crop yield, which leads to higher input and greater soil residue of N fertilizer (Cai, 2019). A study shows that in China, the amount of pure N applied for protected cultivation is several times or even tens of times higher compared to that applied for field cultivation (Du, 2007). Due to the lack of rainwater leaching, a large quantity of fertilizer N remains in the soil, resulting in a series of problems such as soil acidification, salinization, nutrient imbalance, and crop toxicity from nitrite and ammonia, which hinders the sustainable development of protected agriculture.

However, the soil residual fertilizer nitrogen (SRFN) is also a N nutrient resource for the subsequent crops that cannot be ignored. The research of Bhogal (Bhogal et al., 2000) and Dong (Dong et al., 2012) showed that the SRFN reuse rates by subsequent crops could reach 14% and 20%, respectively. The crop reuse of SRFN may be closely related to the amount, location and form of SRFN (Guo et al., 2022; Hong et al., 2021). Research has shown that the crop reuse amount of SRFN increased with the increase in SRFN amount and gradually decreased with increasing planting years (Lu et al., 2013; Yang et al., 2013). Mineralization is an important transformation approach for SRFN in soil. N mineralization promotes the soil organic N converting into mineral N that is preferred by crops (Guntiñas et al., 2012), which is conducive to the crop reuse of more SRFN. Furthermore, the reuse of SRFN may also be related to the absorption capacity of subsequent crops, and it is worth studying how to exert the crop’s biological potential for improving the utilization efficiency of SRFN.

Field management practices play an important role in regulating the fate of SRFN. The partitioning of post-harvest surplus N among subsequent crop recovery, soil organic immobilization, nitrate leaching, and gaseous emissions is strongly modulated by cropping system type and field management choices. Diversified cropping systems and cover crops generally enhance microbial N immobilization, reduce the immediate risk of nitrate leaching, and prolong the residence time of residual N within the soil profile. In contrast, conventional monoculture combined with intensive tillage accelerates the mineralization of legacy residual N and increases the risk of off-field N losses (Ghorbani, 2025). Legume-involved cropping systems generate substantial residual N, the ultimate fate of which depends on subsequent management decisions, including tillage intensity, residue handling, and fallow duration (Desta, 2026). A consecutive ^15^N tracing study on the Loess Plateau demonstrated that diversified cropping systems increased plant N accumulation from residual fertilizer N by 48.3%–255.0% (Wang et al., 2026). Previous studies have shown that the recovery rates of returned straw N in the second season range from 1.60% to 5.51%, with cumulative recovery reaching up to 26% over four consecutive seasons (Wei et al., 2026). In addition, intercropping systems can generate positive legacy effects on subsequent crops (Zhang et al., 2025). In this study, over 70% of SRFN remained in the soil and 2.48%–8.95% was absorbed by subsequent crops, which is consistent with the aforementioned literature, further confirming that field management practices are effective means of regulating the fate of residual fertilizer N in protected agricultural systems.

In protected cultivation, different crop rotation systems provide varied backgrounds for studying nitrogen dynamics. For instance, sequential cultivation of shallow-rooted and deep-rooted crops may create conditions that influence the distribution and potential recovery of SRFN across soil layers. In this context, quantitatively evaluating the reuse of SRFN under different rotation backgrounds is of great practical significance for developing integrated management practices that contribute to the ecological reduction of SRFN.

Fertilization and irrigation are the most common field management practices in protected cultivation (Mahmud et al., 2021). Previously, research on water and nitrogen has mainly focused on in-season applied fertilizer nitrogen (AFN), with the primary goal of improving the AFN use efficiency for the current season (Guo et al., 2022; Hong et al., 2021; Jin et al., 2023; Wang et al., 2022a, b, 2024). Limited attention has been paid to SRFN. Irrigation may affect the N transport and transformation and the crop root development, while fertilization influences the residual state of SRFN. Therefore, fertilization or irrigation may impact the reuse of SRFN by subsequent crops.

This study consisted of two independent experiments designed to test the hypotheses that different nitrogen application rates in the previous season (Experiment I) or different irrigation strategies for the subsequent crop (Experiment II) may affect the reuse of SRFN by subsequent crops. Both experiments were conducted under two crop rotation systems (“pakchoi-pakchoi” and “pakchoi-tomato”), which served as parallel backgrounds. Using ^15^N isotope tracing technology with pakchoi and tomato as plant materials, the objectives were: (1) to quantitatively investigate the effects of previous nitrogen application rate (Experiment I) or subsequent irrigation strategy (Experiment II) on the AFN reuse rate by subsequent crops; (2) to clarify the balance characteristics of SRFN under different fertilization or irrigation regimes; (3) to identify the factors influencing the AFN reuse rate of subsequent crops.

## 2 Material and method

### 2.1 Experimental site

The experiment was conducted from February 2023 to July 2024 at the Old-zone Fruit Orchard Technology Demonstration Base in Yunxiao County (23°57′38″ N, 117°20′5″ E, **Figure 1**). Yunxiao County has a subtropical maritime monsoon climate. The annual average temperature is 21.3 °C. In the past 10 years, the average temperature in January was 13.4 °C, and in July, it was 28.2 °C. The annual precipitation is 1730.6 mm, with a frost-free period of 347 days. Prior to the experiment, the measured soil (ultisols) physical and chemical properties in the 0–20 cm layer of the experimental field were: salt mass fraction of 2.53 g/kg, organic matter of 2.2%, total N of 0.98 g/kg, available P of 15.1 mg/kg, available K of 116.7 mg/kg, bulk density of 1.38 g/cm^3^ and field capacity of 23.1%.

**Figure 1:**
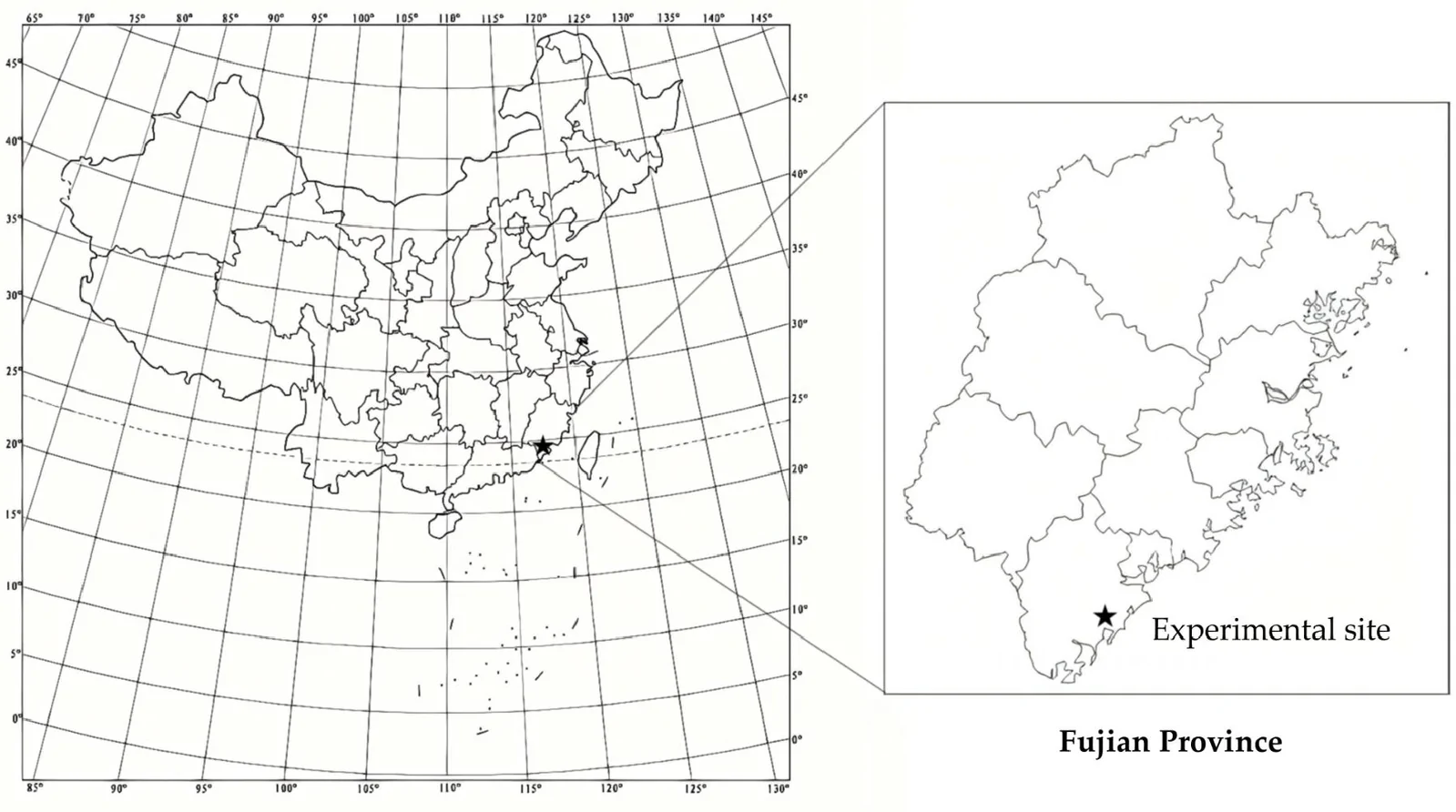
Experimental site (Yunxiao County, Fujian province, China).

### 2.2 Experimental design

This study consisted of two independent experiments conducted in 2023 and 2024, respectively. In both experiments, two crop rotation modes (“pakchoi-pakchoi” and “pakchoi-tomato”) were established in parallel to examine the reuse of SRFN by subsequent crops. Experiment in 2023 investigated the effect of different nitrogen application rates in the previous season, while experiment in 2024 investigated the effect of different irrigation strategies for the subsequent crop. No pooling or repetition structure was assumed between years.

The experiment used pakchoi (*Brassica rapa L. Ssp. chinensis (Hanelt.)*, “Jinpin Aijiao”) and tomato (*Solanum lycopersicum*, “Dahongbao”) as plant materials. In each growing season, the first crop of pakchoi was cultivated from late winter to early spring, with transplanting dates on 15 February 2023 and 9 February 2024, respectively. The whole growth period of pakchoi was approximately 35 days, meaning it was harvested in early spring. For the subsequent crop, pakchoi was grown in spring, with a growth period of approximately 35 days. Tomato, with a whole growth period of approximately 120 days, was cultivated from spring through summer. The fallow period between the first and subsequent crops was set according to local practices, which was lasting 10–15 days. Both pakchoi and tomato were transplanted to the field after seedling growth.

The experiment was conducted in the playhouse, as shown in **Figure 2**. Prior to the experiment, the soil from the 0–60 cm layer was excavated in layers. The soil was then homogenized thoroughly before being backfilled into the 60 cm deep PVC partition in layers, following the original order. The PVC partition divided the experimental area into 36 small blocks, each measuring 1.2 m × 1.2 m. In 2023 and 2024, 18 small blocks were used for each year. In a single block, 9 plants of pakchoi or tomato were cultivated. Each block was equipped with three independent irrigation control valves on the pipes. Irrigation was carried out when the soil moisture content dropped to the designed lower irrigation limit. The water supply method used was drip irrigation.

**Figure 2:**
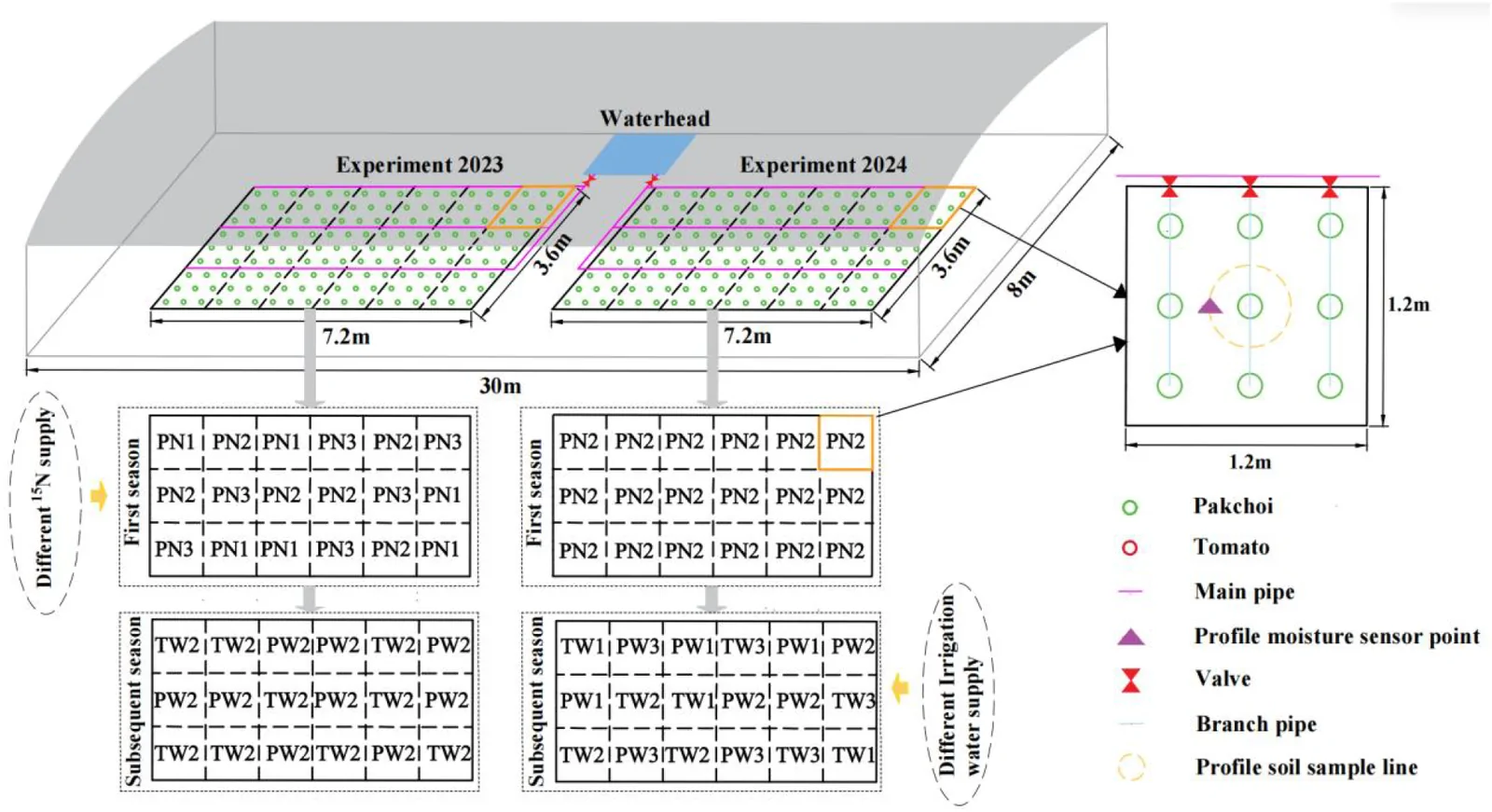
Layout of experimental blocks (in this study, pakchoi was planted in the first crop season, pakchoi or tomato was planted in the subsequent crop season. In 2023, the first season crop was treated with three nitrogen application rates; in 2024, the subsequent crop was treated with three different irrigation lower limits. The experiments in 2023 and 2024 are two independent experiments).

The 2023 experiment was designed to investigate SRFN reuse by subsequent crops under different AFN levels of the previous season. Therefore, the first crop of pakchoi was treated with three AFN levels (80 kg/ha, 120 kg/ha, 160 kg/ha) using ^15^N isotope-labeled urea (abundance of 19.6%) to create three different states of N residue. Because there were two rotation modes, “pakchoi-pakchoi” and “pakchoi-tomato”, the experiment of 2023 consisted of 3 × 2 = 6 treatments. Each treatment has three repetitions. Each repetition occupied one block. The blocks were arranged randomly. When the subsequent crop was pakchoi, the irrigation lower limit for the whole growth period was 70% of field capacity. Owing to the short growth cycle of pakchoi, a uniform lower limit for soil moisture was maintained across all growth stages. The upper limit was 100%. When the subsequent crop was tomato, the irrigation lower limits were 60%-70%-70% (60%, 70% and 70% for the seedling stage, the flowering and fruit-setting stage, and the maturity stage, respectively), with an upper limit of 100%. For the second crop, whether pakchoi or tomato, N fertilization was applied using common urea without ^15^N labeling, with the application rates of 120 kg/ha for pakchoi and 180 kg/ha for tomato. Details are shown in **Tables 1** and **2**.

**Table 1:** Nitrogen application and irrigation design of 2023 under the pakchoi-pakchoi rotation system.

Cultivation sequence	Crop	Type of N fertilizer	N application rate (kg/ha)	Lower irrigation limit (%)	Upper irrigation limit (%)
First crop	Pakchoi	^15^N-labeled urea (^15^NH_2_CO^15^NH_2_)	80 (PN1)	70	100
			120 (PN2)		
			160 (PN3)		
Second crop	Pakchoi	Common urea (NH_2_CONH_2_)	120	70	100

In the table, 70% represents that the irrigation lower limit accounts for 70% of the field capacity, and 100% represents that the irrigation upper limit accounts for 100% of the field capacity. Irrigation starts when the soil moisture in the 0–20 cm soil layer reaches the lower limit and stops when it reaches the upper limit.

**Table 2:** Nitrogen application and irrigation design of 2023 under the pakchoi-tomato rotation system.

Cultivation sequence	Crop	Type of N fertilizer	N application rate (kg/ha)	Lower irrigation limit (%)	Upper irrigation limit (%)
First crop	Pakchoi	^15^N-labeled urea (^15^NH_2_CO^15^NH_2_)	80 (TN1)	70	100
			120 (TN2)		
			160 (TN3)		
Second crop	Tomato	Common urea (NH_2_CONH_2_)	180	60-70-70	100

In the table, 70% represents that the irrigation lower limit accounts for 70% of the field capacity, and 100% represents that the irrigation upper limit accounts for 100% of the field capacity. Further, 60-70-70% represents that the irrigation lower limits are 60%, 70% and 70%, respectively, for the tomato seedling stage, flowering and fruit-setting stage, and maturity stage. Irrigation starts when the soil moisture in the 0–20 cm soil layer reaches the lower limit and stops when it reaches the upper limit.

The 2024 experiment aimed to study the SRFN reuse under different irrigation strategies of the succeeding crop cultivation season. The N application for the first crop, pakchoi, was 120 kg/ha of ^15^N isotope-labeled urea with an abundance of 19.6%. Different irrigation lower limits were designed for the succeeding crops. For pakchoi, the three irrigation lower limits were 60%, 70%, and 80%. For tomatoes, the three irrigation lower limits were 50%-60%-60%, 60%-70%-70% and 70%-80%-80%. The irrigation upper limit was 100% for all treatments. In total, there were six treatments for the 2024 experiment, and each treatment has three replications. Each replication occupied one block. The blocks were arranged randomly. Similarly, for the second crop, the N fertilizer was common urea without ^15^N labeling, applied at rates of 120 kg/ha for pakchoi and 180 kg/ha for tomato. The details are shown in **Tables 3** and **4**.

**Table 3:** Nitrogen application and irrigation design of 2024 under the pakchoi-pakchoi rotation system.

Cultivation sequence	Crop	Type of N fertilizer	N application rate (kg/ha)	Lower irrigation limit (%)	Upper irrigation limit (%)
First crop	Pakchoi	^15^N-labeled urea (^15^NH_2_CO^15^NH_2_)	120	70	100
Second crop	Pakchoi	Common urea (NH_2_CONH_2_)	120	60 (PW1)	100
				70 (PW2)	
				80 (PW3)	

In the table, 70% represents that the irrigation lower limit accounts for 70% of the field capacity, and 100% represents that the irrigation upper limit accounts for 100% of the field capacity. Irrigation starts when the soil moisture in the 0–20 cm soil layer reaches the lower limit and stops when it reaches the upper limit.

**Table 4:** Nitrogen application and irrigation design of 2024 under the pakchoi-tomato rotation system.

Cultivation sequence	Crop	Type of N fertilizer	N application rate (kg/ha)	Lower irrigation limit (%)	Upper irrigation limit (%)
First crop	Pakchoi	^15^N-labeled urea (^15^NH_2_CO^15^NH_2_)	120	70	100
Second crop	Tomato	Common urea (NH_2_CONH_2_)	180	50-60-60 (TW1)	100
				60-70-70 (TW2)	
				70-80-80 (TW3)	

In the table, 70% represents that the irrigation lower limit accounts for 70% of the field capacity, and 100% represents that the irrigation upper limit accounts for 100% of the field capacity. Further, 50-60-60% represents that the irrigation lower limits are 50%, 60% and 60%, respectively, for the tomato seedling stage, flowering and fruit-setting stage, and maturity stage (the representation for other treatments follows the same format). Irrigation starts when the soil moisture in the 0–20 cm soil layer reaches the lower limit and stops when it reaches the upper limit.

To prevent the influence of other nutrient inputs on the experimental results, the application rates of phosphorus and potassium fertilizers were kept the same across all treatments for the same crop. For pakchoi, superphosphate (60 kg/ha P_2_O_5_) and potassium sulfate (90 kg/ha K_2_O) were used, with 40% applied as base fertilizer, 20% applied when the plants has developed four leaves and one central growing point, 20% at the six-leaf stage, and 20% at the eight-leaf stage. For tomato, superphosphate (90 kg/ha P_2_O_5_) and potassium sulfate (120 kg/ha K_2_O) were applied, with the application ratio of 1:1:1 among base fertilization, the first fruit set fertilization, and the second fruit set fertilization. The fertilizer was applied into small holes dug in advance next to the crops. The application rates and methods of phosphorus and potassium fertilizers were based on local practice. The other field management measures, such as pest, disease, and weed control, were consistent across all treatments.

### 2.3 Sampling and measurement

Soil moisture content was measured daily using a profile soil moisture sensor (PR2, Delta-T Devices Ltd, Cambridge, UK) at depths of 0–20 cm, 20–40 cm and 40–60 cm. The measurement probes were placed at the midpoint between two crop plants.

After the second crop cultivation, the soil samples were collected. For each block, a sampling line was designated, with three sampling points within the line. A twisted soil auger was used to collect soil samples from the 0–20 cm, 20–40 cm, and 40–60 cm soil layers. After collection, soil samples from the same soil layer were mixed evenly. A portion of the fresh samples was used to observe the SRFN forms (mineral ^15^N and organic ^15^N), while another portion was air-dried, ground, and sieved for the determination of total ^15^N.

During the crop harvest period, plant samples were collected by randomly selecting three plants from each block. For pakchoi, the roots were washed clean and then separated from the above-ground portion using a knife. Similarly, for tomatoes, the roots, stems, leaves, and fruits were separated. Different parts of the plants were placed in an oven to deactivate enzymes at 105 °C for 30 min and then dried at 75 °C until a constant weight was achieved. The dried samples were ground into powder and passed through a 100-mesh sieve to measure the total N content and the ^15^N atom per cent excess in the plants.

The content of total N in the plants was measured using the Kjeldahl method after digestion with H_2_SO_4_-H_2_O_2_. The extraction method for mineral N in fresh soil samples followed the procedure described by Xu (Xu et al., 2020). The ^15^N atom percent excess in soil and plant samples was determined using a mass spectrometer (Finnigan-Mat-251, Mass-Spectrometers, Finnigan Company, Bremen. Germany).

### 2.4 Calculations

In this study, the AFN reuse efficiency is defined as the ratio of the ^15^N amount absorbed by the subsequent crop to the ^15^N amount applied in the first crop. The calculation method is as follows (Equations 1, 2; Hou et al., 2023):

(1) $$N\text{dff}={\text{C}}_{\text{s}}\times \frac{{\text{E}}_{\text{s}}}{{\text{E}}_{\text{f}}}$$

(2) $$NUE=(\;\frac{N\text{dff}}{{\text{M}}_{\text{f}}}\;)\times 100\%$$

where *Ndff* represents the amount of plant N derived from the ^15^N-labeled fertilizer (kg/ha), *C_s_* is the content of total N in the plant sample (kg/ha), *E_s_* is the ^15^N atom percent excess in the plant sample (%), *E_f_* is the ^15^N atom percent excess in the labeled fertilizer (%), and *M_f_* is the amount of AFN (%).

The soil organic ^15^N content is the difference between the total ^15^N content and the mineral ^15^N content.

The ^15^N loss, a balance value and not a direct measurement of leaching or emissions, might include gaseous losses, deep leaching losses, and other unknown pathways. In this study, the ^15^N loss is calculated as the total ^15^N supply minus the ^15^N recovery. The ^15^N recovery is the sum of the ^15^N absorbed by the plants, and the total ^15^N remained in the 0–60 cm soil layer (Hou et al., 2017).

The ^15^N loss rate is the ratio of the ^15^N loss to the total applied ^15^N in the first crop.

### 2.5 Data analysis

Data were analyzed separately for Experiment of 2023 and Experiment of 2024 using SPSS 17.0 software. For each experiment, soil ^15^N data from the two rotation systems were analyzed independently using one-way analysis of variance (one-way ANOVA), as the crop rotation mode was treated as a parallel experimental background rather than a statistical factor. Duncan’s multiple range test was conducted as a *post-hoc* procedure to compare treatment means. All results are presented as mean ± standard deviation.

## 3 Results

### 3.1 Effects of different irrigation strategies on moisture content in soil profile

During the subsequent crop cultivation, the soil moisture content at 0–20 cm depth showed the most dynamic changes throughout the entire growth period of the pakchoi or tomato, followed by 20–40 cm depth, while the variation at 40–60 cm depth was the smallest (**Figures 3a–f**). The differences in average soil moisture content over the entire growth period under different irrigation strategies were mainly observed at the 0–20 cm and 20–40 cm soil depths. At the 0–20 cm depth, the average soil moisture contents for PW1, PW2, and PW3 were 25.5%, 26.9% and 28.7%, respectively, with significant (*p* < 0.05) differences among treatments; similarly, significant (*p* < 0.05) differences were also observed among TW1 (25.1%), TW2 (26.8%), and TW3 (28.3%). For the 20–40 cm depth, the treatment differences in soil moisture content among PW1, PW2 and PW3 followed a similar pattern to those at the 0–20 cm depth, but no significant differences were found between TW2 and TW3. At the 40–60 cm depth, there were almost no differences in the average soil moisture content across different treatments, with pakchoi cultivation soil and tomato cultivation soil having moisture contents of 27.5–28.4% and 24.5–24.7%, respectively. However, it was worth noting that the fluctuation in soil moisture at the 40–60 cm depth was more pronounced in the tomato cultivation soil than in the pakchoi soil. Overall, the results indicated that the higher irrigation limits are beneficial for maintaining higher moisture content in the shallow and middle soil layers throughout the crop growth period.

**Figure 3:**
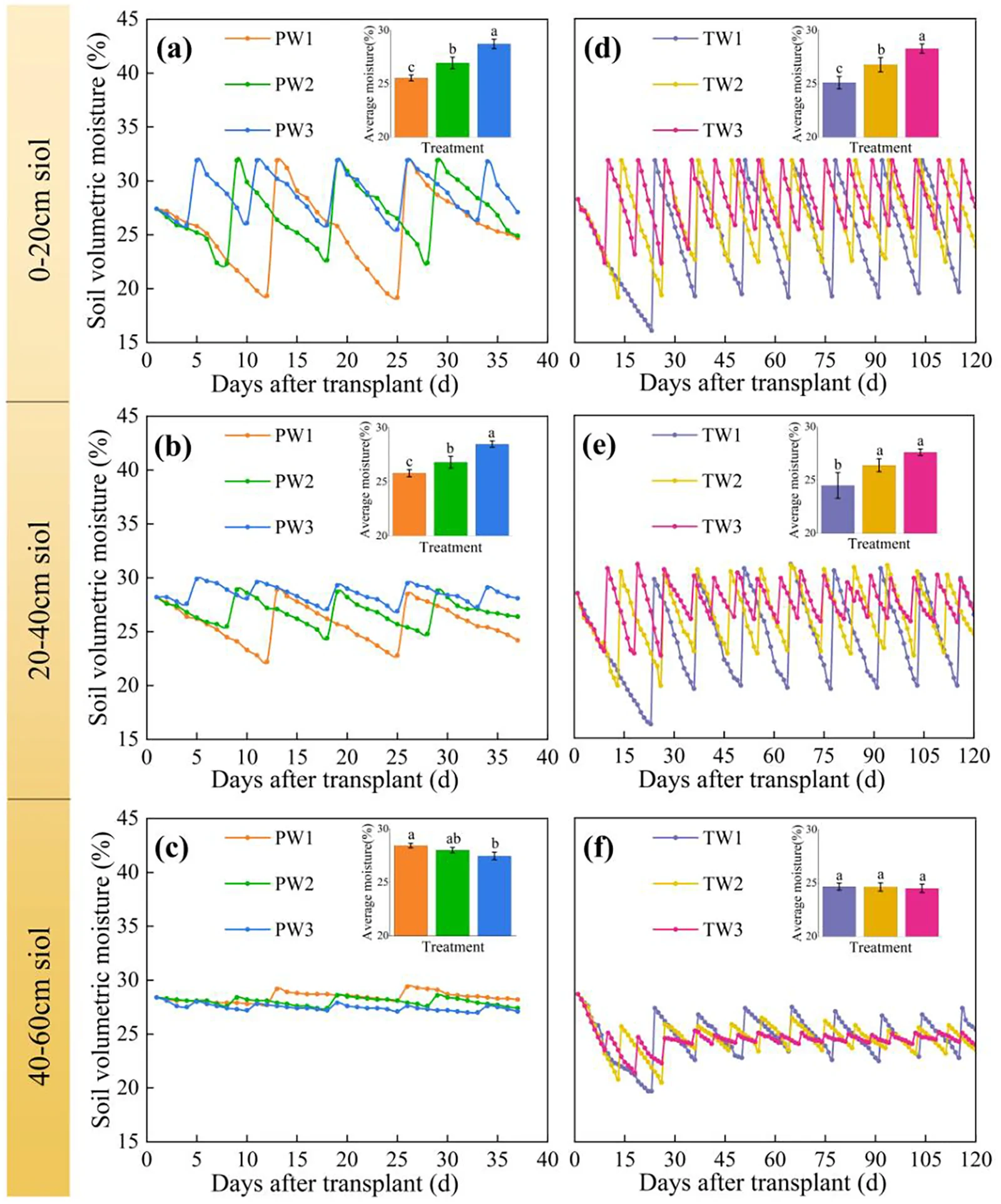
The effect of different irrigation strategies on the moisture content in the soil profile **(a, c, e)** show soil moisture at 0–20, 20–40, and 40–60 cm depths when pakchoi was the subsequent crop; **(b, d, f)** show the same depths when tomato was the subsequent crop. The bar charts indicate the mean moisture content over the entire growth period for each layer. Different letters indicate significant differences at *p* < 0.05 (Duncan’s test). P and T denote pakchoi and tomato as subsequent crops; W1, W2, and W3 represent low, medium, and high irrigation lower limits (2024 experiment). N1–N3 refer to previous N rates (2023 experiment). Values are means ± SD.

### 3.2 Amount and form of SRFN (^15^N) as influenced by N application rates or irrigation strategies under different crop rotations

In the experiment of 2023, the amount of applied fertilizer nitrogen (AFN) in the first crop significantly influenced the residual ^15^N in soil layers after cultivation of the subsequent crop. A higher AFN rate led to greater total ^15^N residue, and correspondingly, higher residual amounts of both mineral and organic ^15^N (**Figures 4a, b**).

**Figure 4:**
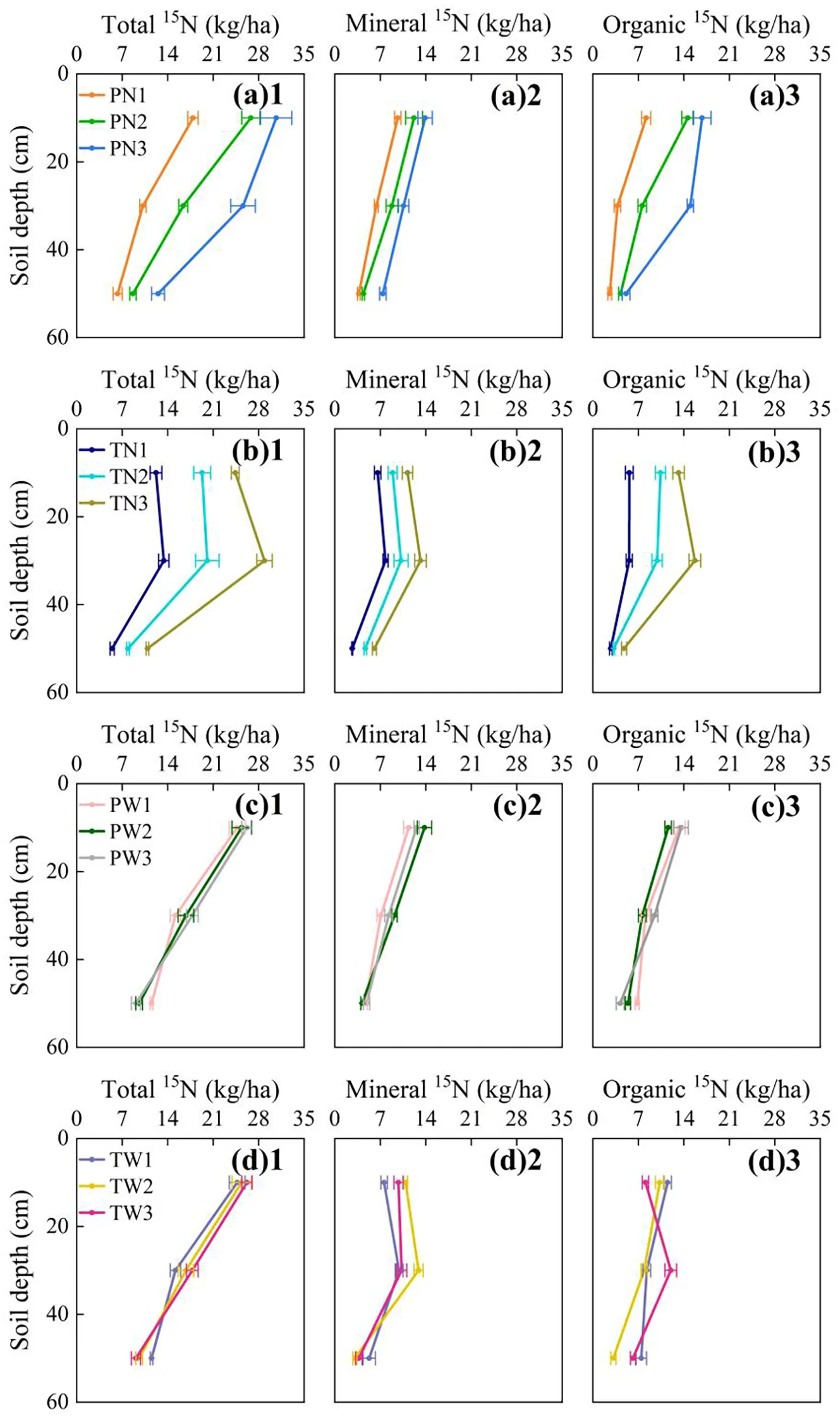
The soil total ^15^N, mineral ^15^N, and organic ^15^N in the soil profile after the cultivation of the succeeding crop influenced by N application rates or irrigation strategies under different crop rotations [**(a)1–(a)3** and **(b)1–(b)3** are from the 2023 experiment (three previous N rates: N1 = 80, N2 = 120, N3 = 160 kg/ha) for subsequent pakchoi **(a)** and tomato **(b)**. **(c)1–(c)3** and **(d)1–(d)3** are from the 2024 experiment (three irrigation lower limits: W1 = low, W2 = medium, W3 = high) for subsequent pakchoi **(c)** and tomato **(d)**. In each panel group, subscripts 1–3 denote total ^15^N, mineral ^15^N, and organic ^15^N, respectively. Values are means ± SD].

Under the pakchoi-pakchoi rotation background, the total ^15^N residue in the 0–20 cm soil layer ranged from 17.9 to 30.7 kg/ha, with mineral ^15^N accounting for 45% to 54% of the total ^15^N and ranging from 9.7 to 13.9 kg/ha. Under the pakchoi-tomato rotation background, the total ^15^N residue in the 0–20 cm soil layer ranged from 12.2 to 24.4 kg/ha, with mineral ^15^N residue ranging from 6.6 to 11.2 kg/ha. In both rotation backgrounds, a lower amount of soil residual fertilizer nitrogen (SRFN) resulted in a higher proportion of mineral ^15^N, a trend observed in both the 0–20 cm and 20–40 cm soil layers.

The cultivation of subsequent crops altered the profile distribution of SRFN. In the pakchoi-tomato rotation background, total ^15^N residue in the 0–20 cm and 40–60 cm layers showed different patterns compared to the pakchoi-pakchoi background, with distinct distribution characteristics in the 20–40 cm layer.

In the experiment of 2024, the AFN rate in the first crop was fixed at 120 kg N/ha, and different irrigation strategies were applied to the subsequent crop. The total ^15^N residue in the 0–20 cm soil layer ranged from 24.7 to 26.2 kg/ha under the pakchoi-pakchoi rotation background and from 17.9 to 21.2 kg/ha under the pakchoi-tomato rotation background (**Figures 4c, d**). For the 20–40 cm soil layer, the total ^15^N residue showed different patterns between the two rotation backgrounds, with corresponding differences in mineral ^15^N content. In the 40–60 cm soil layer, total ^15^N and mineral ^15^N showed limited variation across treatments in both rotation backgrounds.

Under the pakchoi-pakchoi rotation background, a higher irrigation lower limit for the subsequent crop resulted in slightly higher total ^15^N residue in the 0–20 cm or 20–40 cm layer but lower total ^15^N residue in the 40–60 cm soil layer (**Figure 4c**), indicating that irrigation with high frequency but small quota helped retain more SRFN in the shallow and middle soil layers. Under the pakchoi-tomato rotation background, the highest amount of total ^15^N residue in the 0–20 cm soil layer was detected under TW2, reaching 21.2 kg/ha, while the highest in the 20–40 cm soil layer was under TW3 (22.3 kg/ha) (**Figure 4d**). In both rotation backgrounds, the greatest amount of mineral ^15^N was found in the W2 irrigation lower limit for the 0–20 cm or 20–40 cm soil layer.

**Table 5** presents the effects of previous fertilization (experiment of 2023) and subsequent irrigation (experiment of 2024) on total ^15^N, mineral ^15^N, and organic ^15^N in different soil layers. As shown in the table, in the experiment of 2023, the AFN profoundly influenced total ^15^N, mineral ^15^N, and organic ^15^N in different soil layers of the subsequent crop season, with these effects reaching a highly significant level in both rotation backgrounds. In the experiment of 2024, irrigation had a significant effect on mineral ^15^N in the 0–40 cm layer and total ^15^N in the 20–60 cm layer, but had no significant effect on total ^15^N in the 0–20 cm layer in either rotation background.

**Table 5:** Effects of previous fertilization (N) or subsequent irrigation (W) on total ^15^N, mineral ^15^N, and organic ^15^N in different soil layers.

Year	Soil layer	Crop rotation mode	Treatment	Total ^15^N	Mineral ^15^N	Organic ^15^N
2023	0–20 cm	Pakchoi-pakchoi	N	**	**	**
		Pakchoi-tomato	N	**	**	**
	20–40 cm	Pakchoi-pakchoi	N	**	**	**
		Pakchoi-tomato	N	**	**	**
	40–60 cm	Pakchoi-pakchoi	N	**	**	**
		Pakchoi-tomato	N	**	**	**
2024	0–20 cm	Pakchoi-pakchoi	W	ns	*	ns
		Pakchoi-tomato	W	ns	**	**
	20–40 cm	Pakchoi-pakchoi	W	*	**	*
		Pakchoi-tomato	W	**	**	**
	40–60 cm	Pakchoi-pakchoi	W	**	ns	**
		Pakchoi-tomato	W	**	*	**

In the table, **, *, and ns indicate that the experimental treatment has a highly significant (*p* < 0.01), significant (*p* < 0.05), or non-significant (ns) effect on the parameter, respectively.

### 3.3 Biomass accumulation and ^15^N reuse of the subsequent crop as influenced by N application rates or irrigation strategies under different crop rotations

An increased amount of AFN in the first crop from N1 to N2 did not have a significant effect on the biomass accumulation of the subsequent pakchoi. However, when the AFN was increased from N1 to N3, the biomass of the subsequent pakchoi significantly (*p* < 0.05) increased, with the increase observed in both the aboveground and underground parts (**Table 6**). In contrast, under the three AFN rates, no significant difference was found in the biomass of the subsequent tomato (*p >*0.05), with similar regularity observed in various organs, including leaves, roots, and fruits.

**Table 6:** The biomass of different organs of the subsequent crop as influenced by N application rates or irrigation strategies under different crop rotations.

Year	Treatment	Whole plant (kg/ha)	Leaf		Root		Stem		Fruit	
			Biomass (kg/ha)	Proportion (%)	Biomass (kg/ha)	Proportion (%)	Biomass (kg/ha)	Proportion (%)	Biomass (kg/ha)	Proportion (%)
2023	PN1	1201.77 ± 69.88 b	1172.83 ± 68.03 b	97.59 ± 0.02 a	28.93 ± 1.85 b	2.41 ± 0.02 c	--	--	--	--
	PN2	1390.60 ± 104.11 ab	1354.47 ± 101.01 ab	97.40 ± 0.03 b	36.13 ± 3.10 a	2.60 ± 0.03 b	--	--	--	--
	PN3	1408.17 ± 106.01 a	1370.83 ± 103.17 a	97.35 ± 0.02 c	37.33 ± 2.86 a	2.65 ± 0.02 a	--	--	--	--
	TN1	9634.80 ± 207.29 a	4821.33 ± 46.13 a	50.05 ± 0.63 a	289.37 ± 10.25 a	3.00 ± 0.04 b	1314.10 ± 42.55 b	13.64 ± 0.16 b	3210.00 ± 110.41 a	33.31 ± 0.43 b
	TN2	9915.37 ± 379.27 a	4872.70 ± 184.74 a	49.14 ± 0.03 b	306.37 ± 15.95 a	3.09 ± 0.04 a	1386.23 ± 46.99 ab	13.98 ± 0.06 ab	3350.07 ± 131.61 a	33.79 ± 0.04 ab
	TN3	10,019.43 ± 411.34 a	4889.53 ± 225.50 a	48.79 ± 0.25 b	313.53 ± 11.74 a	3.13 ± 0.02 a	1412.10 ± 31.45 a	14.10 ± 0.27 a	3404.27 ± 142.84 a	33.98 ± 0.06 a
2024	PW1	1091.30 ± 46.53 b	1064.37 ± 43.36 b	97.54 ± 0.20 b	26.93 ± 3.27 b	2.46 ± 0.20 a	--	--	--	--
	PW2	1404.27 ± 142.07 a	1368.03 ± 138.42 a	97.42 ± 0.01 b	36.23 ± 3.65 a	2.58 ± 0.01 a	--	--	--	--
	PW3	1269.77 ± 72.56 ab	1241.60 ± 70.75 ab	97.78 ± 0.02 a	28.17 ± 1.80 b	2.22 ± 0.02 b	--	--	--	--
	TW1	8930.25 ± 406.04 b	4571.11 ± 102.68 b	51.22 ± 1.20 a	242.09 ± 18.05 b	2.71 ± 0.08 b	1425.13 ± 52.41 b	15.96 ± 0.16 a	2691.93 ± 233.88 b	30.11 ± 1.26 b
	TW2	9975.11 ± 534.66 a	4748.24 ± 169.92 b	47.63 ± 0.87 b	317.23 ± 36.18 a	3.17 ± 0.19 a	1510.28 ± 39.07 ab	15.16 ± 0.4 3b	3399.36 ± 290.68 a	34.04 ± 0.09 a
	TW3	9557.69 ± 318.32 ab	5023.56 ± 123.94 a	52.57 ± 0.59 a	183.90 ± 17.97 c	1.92 ± 0.12 c	1555.26 ± 55.06 a	16.27 ± 0.04 a	2794.98 ± 128.13 b	29.24 ± 0.46 b

Data are from two independent experiments in 2023 (three previous N rates: N1 = 80, N2 = 120, N3 = 160 kg/ha) and 2024 (three irrigation lower limits: W1 = low, W2 = medium, W3 = high), under pakchoi-pakchoi and pakchoi-tomato rotations. P and T denote pakchoi and tomato as subsequent crops. Values are means ± SD. Different letters indicate significant differences at *p* < 0.05 (Duncan’s test).

Among the three irrigation strategies, W2 has the most pronounced effect on improving the biomass of the entire pakchoi plant, as well as its leaves or roots. Compared to PW1, the total biomass of the entire plant (1404.2 kg/ha), leaf (1368.0 kg/ha), or root (36.2 kg/ha) in PW2 was significantly (*p* < 0.05) higher but was not significantly different when compared to PW3. For tomato, the TW2 treatment showed significantly (*p* < 0.05) higher biomass of root or fruit in comparison to the other two treatments, and the biomass accumulation of the entire plant was also at the highest level.

When the AFN by the first crop increased from N1 to N2, whether the whole plant ^15^N or the organ accumulated, ^15^N accumulation showed a significant (*p* < 0.05) increase for both pakchoi and tomato (**Table 7**). However, when AFN increased from N2 to N3, this increase was not significant. The highest ^15^N absorption amounts of the entire plant for pakchoi and tomato were observed in PN3 and TN3, reaching 3.97 kg/ha and 10.46 kg/ha, respectively. Under the three irrigation strategies, the PW2 treatment resulted in the highest ^15^N absorption amount in the entire plant and various organs of pakchoi. A similar regularity was also found in tomato, where TW2 led to a significantly (*p* < 0.05) higher ^15^N amount in the tomato entire plant, leaves, roots, stems or fruits, with values of 10.74, 4.52, 0.54, 0.92, and 4.76 kg/ha, respectively.

**Table 7:** The ^15^N absorption amount by different organs of the subsequent crop as influenced by N application rates or irrigation strategies under different crop rotations.

Year	Treatment	^15^N in the Whole plant (kg/ha)	Leaf		Root		Stem		Fruit	
			^15^N Amount (kg/ha)	Proportion (%)	^15^N Amount (kg/ha)	Proportion (%)	^15^N Amount (kg/ha)	Proportion (%)	^15^N Amount (kg/ha)	Proportion (%)
2023	PN1	3.03 ± 0.07 b	2.82 ± 0.07 b	93.23 ± 0.23 a	0.20 ± 0.00 b	6.77 ± 0.23 a	--	--	--	--
	PN2	3.88 ± 0.12 a	3.62 ± 0.14 a	93.33 ± 0.61 a	0.26 ± 0.01 a	6.67 ± 0.61 a	--	--	--	--
	PN3	3.97 ± 0.22 a	3.68 ± 0.22 a	92.73 ± 0.60 a	0.29 ± 0.02 a	7.27 ± 0.60 a	--	--	--	--
	TN1	7.09 ± 0.14 b	2.99 ± 0.08 b	42.21 ± 0.40 a	0.35 ± 0.01 b	4.98 ± 0.10 a	0.61 ± 0.01 b	8.67 ± 0.05 a	3.13 ± 0.05 b	44.16 ± 0.42 a
	TN2	10.33 ± 0.72 a	4.33 ± 0.33 a	41.98 ± 0.33 a	0.53 ± 0.04 a	5.12 ± 0.06 a	0.89 ± 0.06 a	8.63 ± 0.06 a	4.57 ± 0.29 a	44.27 ± 0.26 a
	TN3	10.46 ± 0.42 a	4.46 ± 0.23 a	42.66 ± 0.57 a	0.53 ± 0.02 a	5.04 ± 0.03 a	0.90 ± 0.04 a	8.60 ± 0.05 a	4.57 ± 0.12 a	43.70 ± 0.66 a
2024	PW1	3.85 ± 0.09 c	3.56 ± 0.10 c	92.43 ± 0.32 a	0.29 ± 0.01 b	7.57 ± 0.32 a	--	--	--	--
	PW2	4.86 ± 0.14 a	4.51 ± 0.16 a	92.93 ± 0.64 a	0.34 ± 0.02 a	7.07 ± 0.64 a	--	--	--	--
	PW3	4.41 ± 0.12 b	4.09 ± 0.11 b	92.77 ± 0.15 a	0.32 ± 0.02 ab	7.23 ± 0.15 a	--	--	--	--
	TW1	8.18 ± 0.10 c	3.52 ± 0.10 b	43.05 ± 0.74 a	0.42 ± 0.01 b	5.12 ± 0.12 a	0.73 ± 0.04 b	8.87 ± 0.40 a	3.51 ± 0.06 c	42.96 ± 1.13 a
	TW2	10.74 ± 0.22 a	4.52 ± 0.17 a	42.10 ± 0.91 a	0.54 ± 0.03 a	5.00 ± 0.14 a	0.92 ± 0.02 a	8.59 ± 0.14 ab	4.76 ± 0.06 a	44.31 ± 0.92 a
	TW3	8.80 ± 0.32 b	3.78 ± 0.21 b	42.97 ± 1.70 a	0.45 ± 0.02 b	5.15 ± 0.06 a	0.72 ± 0.03 b	8.16 ± 0.05 b	3.85 ± 0.18 b	43.71 ± 1.66 a

Data are from two independent experiments in 2023 (three previous N rates: N1 = 80, N2 = 120, N3 = 160 kg/ha) and 2024 (three irrigation lower limits: W1 = low, W2 = medium, W3 = high), under pakchoi-pakchoi and pakchoi-tomato rotations. P and T denote pakchoi and tomato as subsequent crops. Values are means ± SD. Different letters indicate significant differences at *p* < 0.05 (Duncan’s test).

### 3.4 ^15^N balance and distribution

The AFN rates in the first crop significantly affected the SRFN. After the cultivation of the first crop, a total of 40.0–83.2 kg/ha of ^15^N remained in the soil, showing significant (*p* < 0.05) differences among treatments (**Table 8**). After the cultivation of the subsequent crop, 34.4–68.8 kg/ha and 31.1–64.2 kg/ha of ^15^N remained in the soil for pakchoi and tomato, respectively; 3.0–4.0 kg/ha and 7.1–10.5 kg/ha were absorbed by the subsequent crops for pakchoi and tomato, respectively; and 2.5–10.4 kg/ha and 1.8–8.5 kg/ha were lost from the pakchoi-pakchoi and pakchoi-tomato systems, respectively.

**Table 8:** The ^15^N balance after the subsequent crop season as influenced by N application rates or irrigation strategies under different crop rotations.

Year	Treatment	Total ^15^N (kg/ha)	Soil ^15^N (kg/ha)	Plant ^15^N (kg/ha)	^15^N Loss (kg/ha)
2023	PN1	39.97 ± 2.28 c	34.41 ± 2.44 c	3.03 ± 0.07 b	2.54 ± 0.10 c
	PN2	62.88 ± 4.15 b	51.87 ± 4.80 b	3.88 ± 0.12 a	7.12 ± 0.57 b
	PN3	83.20 ± 5.79 a	68.82 ± 6.36 a	3.97 ± 0.22 a	10.41 ± 0.37 a
	TN1	39.97 ± 2.28 c	31.07 ± 2.54 c	7.09 ± 0.14 b	1.80 ± 0.12 c
	TN2	62.88 ± 4.15 b	47.31 ± 5.10 b	10.32 ± 0.72 a	5.25 ± 0.38 b
	TN3	83.20 ± 5.79 a	64.20 ± 6.16 a	10.46 ± 0.42 a	8.54 ± 0.06 a
2024	PW1	61.70 ± 4.16 a	51.43 ± 4.12 a	3.85 ± 0.09 c	6.42 ± 0.10 a
	PW2	61.70 ± 4.16 a	51.81 ± 4.48 a	4.86 ± 0.14 a	5.03 ± 0.25 b
	PW3	61.70 ± 4.16 a	53.11 ± 4.14 a	4.41 ± 0.12 b	4.18 ± 0.12 c
	TW1	61.70 ± 4.16 a	50.06 ± 4.13 a	8.18 ± 0.10 c	3.46 ± 0.07 a
	TW2	61.70 ± 4.16 a	48.26 ± 4.30 a	10.74 ± 0.22 a	2.70 ± 0.03 b
	TW3	61.70 ± 4.16 a	50.08 ± 4.19 a	8.80 ± 0.32 b	2.82 ± 0.09 b

This study consisted of two independent experiments conducted in 2023 (three previous N rates: N1–N3) and 2024 (three irrigation lower limits: W1–W3), respectively, under two crop rotation modes (pakchoi-pakchoi and pakchoi-tomato). In the treatment labels, P and T denote pakchoi and tomato as subsequent crops, respectively. Values are means ± SD. Different letters in the same column indicate significant differences at *p < 0.05* (Duncan’s test).

The different irrigation strategies for the subsequent crop has no significant effect on the secondary residue of the total soil ^15^N (**Table 8**). Still, irrigation significantly (*p* < 0.05) affected the crop ^15^N re-absorption or ^15^N loss, except that no significant differences in ^15^N loss were found between TW3 and TW2.

Regarding the fate of SRFN from the first crop in the subsequent crop season, 80.3–86.0% of the ^15^N remained in the soil for the pakchoi-pakchoi system, and 72.3–79.8% remained in the soil for the pakchoi-tomato system (**Figure 5**). When pakchoi was cultivated as the subsequent crop, 5.4-8.6% of the SRFN was reused, and when tomato was cultivated, 13.9-19.1% of the SRFN was reused. Increasing the AFN rate in the first crop reduced the proportion of the ^15^N absorbed by the subsequent crop and increased the proportion of ^15^N loss. The W2 irrigation strategy has a significant effect on increasing the proportion of plant ^15^N for both pakchoi and tomato but no significant effect on the proportion of ^15^N loss.

**Figure 5:**
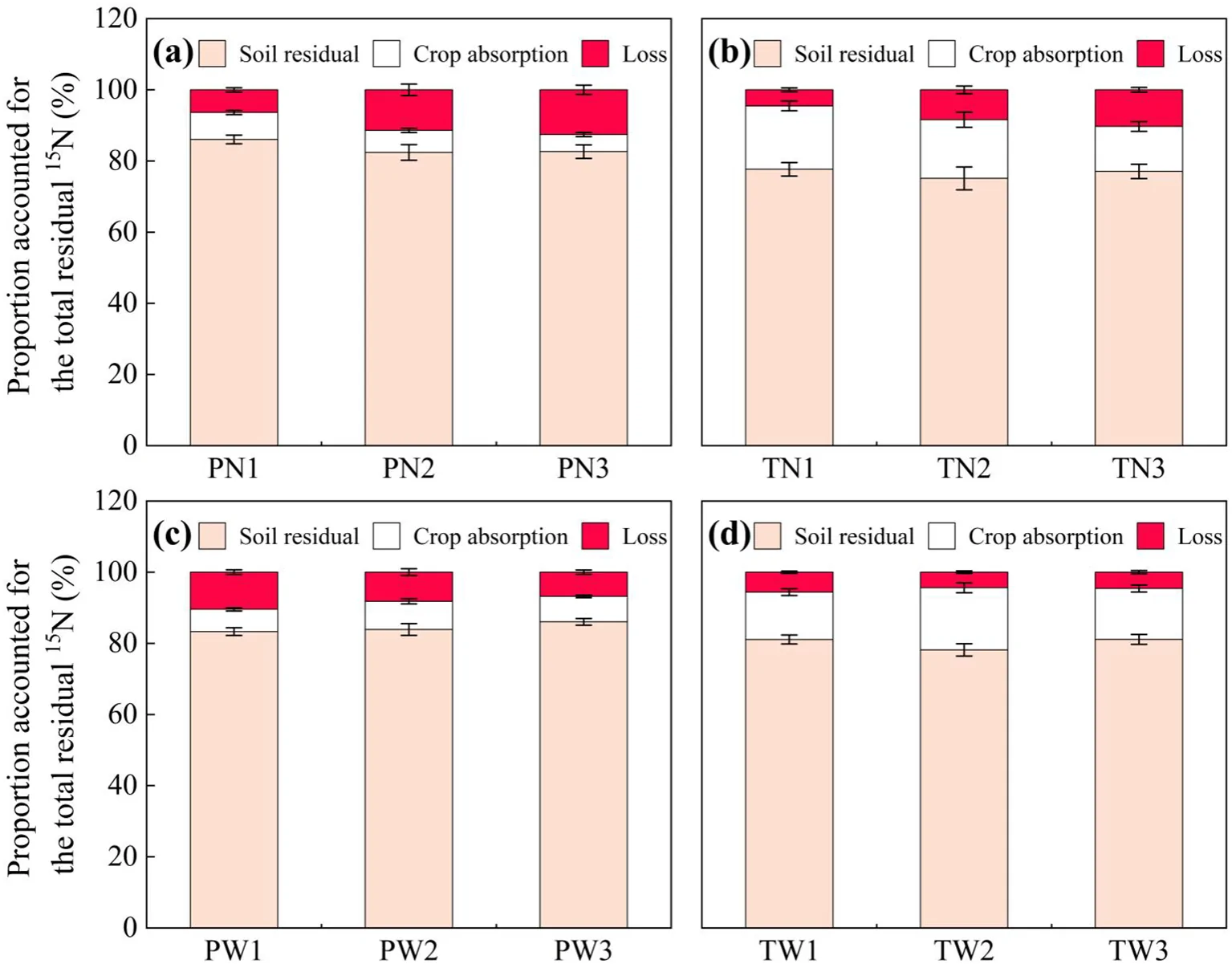
Proportion of ^15^N soil residue, ^15^N plant uptake, and ^15^N loss in the subsequent crop cultivation process relative to the total soil residual ^15^N from the first crop [**(a, b)** are from the 2023 experiment (three previous N rates), with subsequent crops being pakchoi **(a)** and tomato **(b)**. **(c, d)** are from the 2024 experiment (three irrigation lower limits), with subsequent crops being pakchoi **(c)** and tomato **(d)**. Values are means ± SD].

### 3.5 ^15^N reuse rate and ^15^N loss rate

Under different AFN rates in the first crop, the ^15^N reuse rate ranged from 2.48% to 3.79% for the subsequent pakchoi and 6.54% to 8.87% for the tomato (**Figures 6a, b**). The higher AFN rate resulted in the lower ^15^N rreuserate by the subsequent crop. For pakchoi, when AFN increased from N1 to N2 or from N2 to N3, a significant (*p* < 0.05) decrease in the ^15^N reuse rate was observed. For tomatoes, a significant (*p* < 0.05) decrease in the ^15^N reuse rate was observed when AFN increased from N2 to N3. Under different irrigation strategies, W2 most significantly improved the ^15^N reuse rate. Under W2, the re-use rate for pakchoi and tomato reached 4.05% and 8.95%, respectively (**Figures 6c, d**).

**Figure 6:**
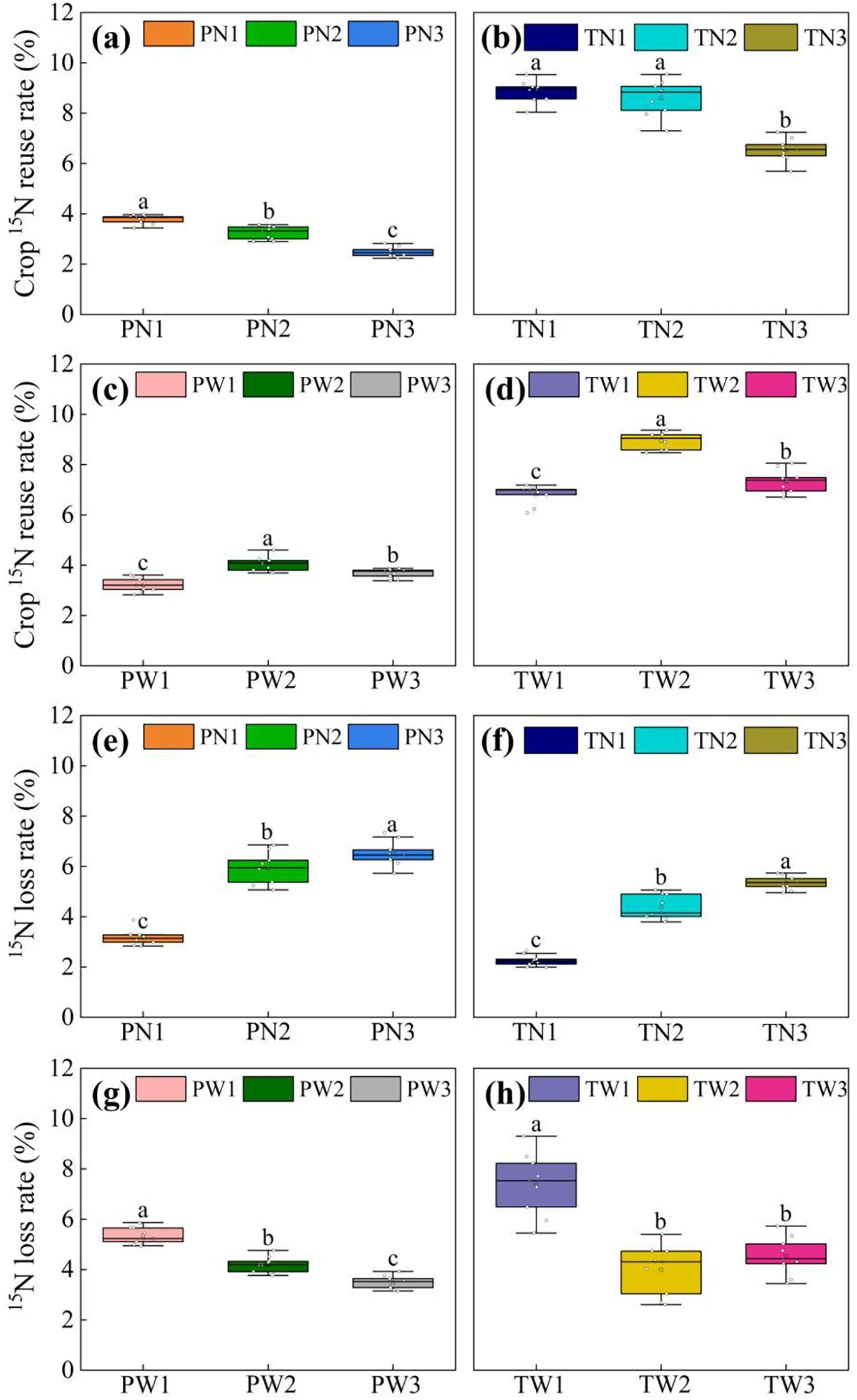
Reuse **(a–d)** and loss **(e–h)** rates of ^15^N under different nitrogen application rates or irrigation strategies under different crop rotations [**(a, b, e, f)**: 2023 experiment with three previous N rates (N1–N3); **(c, d, g, h)**: 2024 experiment with three irrigation lower limits (W1–W3). Subsequent crops: pakchoi (P) in **(a, c, e, g)** and tomato (T) in **(b, d, f, h)**. Values are means ± SD. Different letters indicate significant differences at *p < 0.05* (Duncan’s test). Box plots: box = 25th–75th percentiles, line = median, whiskers = min/max, circles = individual values].

When AFN in the first crop increased (from N1 to N2 and from N2 to N3), the 15N loss rate during subsequent crop cultivation increased significantly (*p* < 0.05), regardless of crop type (**Figures 6e, f**). The highest loss rates for the pakchoi-pakchoi system and the pakchoi-tomato system were observed under PN3 and TN3, reaching 6.5% and 5.3%, respectively. Among the three irrigation treatments, W1 increased the ^15^N loss rate for both pakchoi and tomato (**Figures 6g, h**). The 15N loss rates under PW1 and TW1 were 5.35% and 2.88%, respectively, which were significantly (*p* < 0.05) the highest among their respective treatments.

### 3.6 Correlation between ^15^N reuse and potential impact factors

Under different SRFN from the first crop, the ^15^N reuse rate by the subsequent crop showed a negative correlation with the contents of the total soil ^15^N, the ^15^N in each soil layer, and the mineral ^15^N. However, the crop ^15^N reuse amount was significantly (*p* < 0.01) positively correlated with total ^15^N in the 0–20 cm soil layer, mineral ^15^N in the 0–20 cm layer, and mineral ^15^N in the 20–40 cm layer. For pakchoi, the correlation coefficients were 0.81, 0.86, and 0.82, respectively; for tomato, they were 0.81, 0.86, and 0.83, respectively (**Figures 7a, b**).

**Figure 7:**
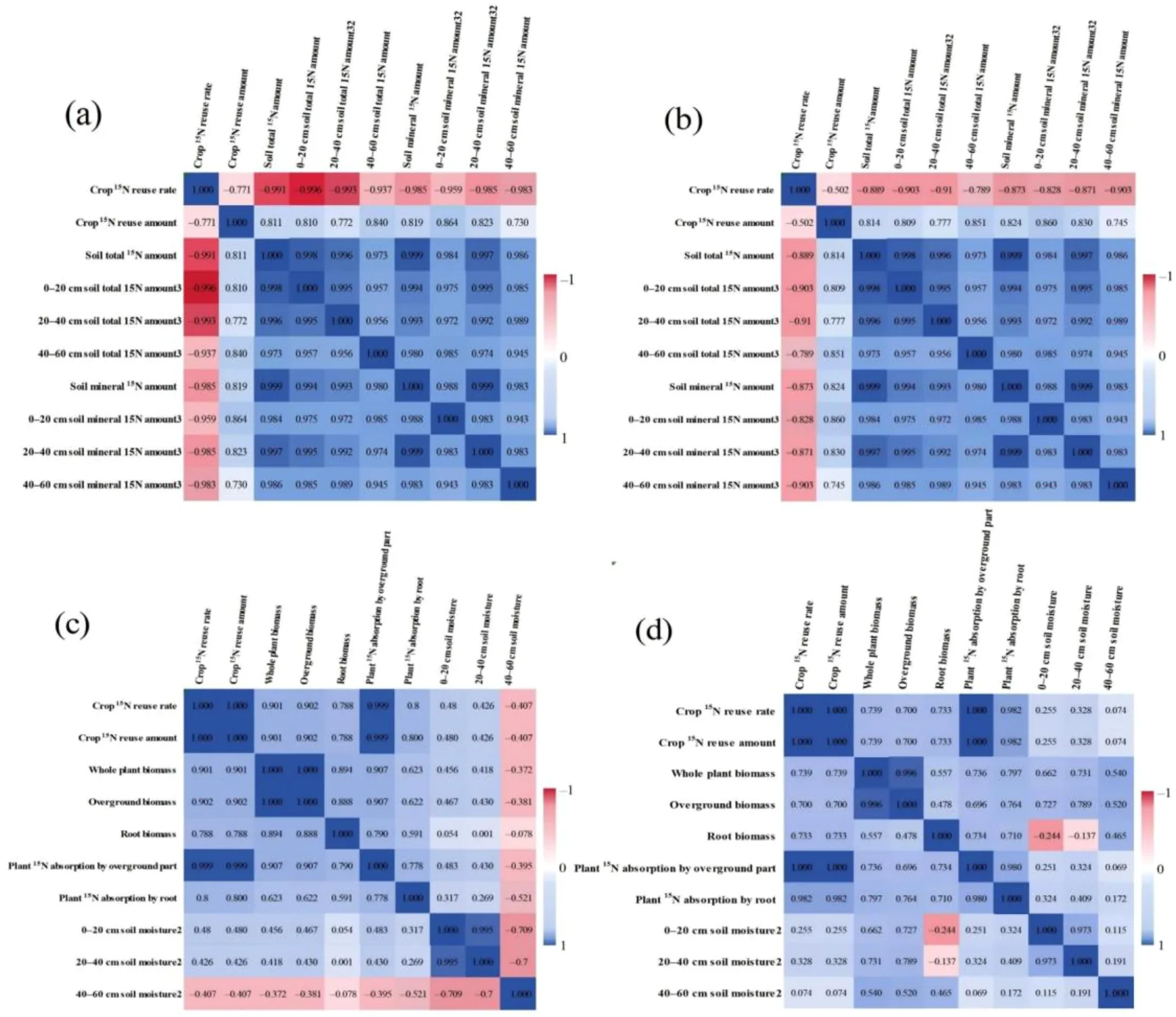
Correlation analysis between ^15^N reuse and possible influencing factors according to Pearson’s correlation coefficient [**(a, b)** are from the 2023 experiment investigating the effects of three previous nitrogen application rates (N1, N2, N3) on ^15^N reuse by subsequent pakchoi **(a)** and tomato **(b)**. **(c,d)** are from the 2024 experiment investigating the effects of three irrigation lower limits (W1, W2, W3) on ^15^N reuse by subsequent pakchoi **(c)** and tomato **(d)**. Values are means ± SD].

When the SRFN condition was the same, the ^15^N reuse rate by the subsequent crop showed a significant (*p* < 0.05) positive correlation with the root biomass (**Figures 7c, d**). The correlation coefficient was 0.79 for pakchoi and 0.73 for tomato.

## 4 Discussion

The main research question addressed in this study is related to understanding the behavior of SRFN in soil under different fertilization levels or irrigation regimes under crop rotation systems. Fertilization according to soil fertility and crop nutrient requirements represents one of the fundamental principles of fertilizer management; however, despite the application of such practices, considerable amounts of SRFN often remain in the soil. In this context, the results of the study contribute to a better understanding of SRFN dynamics and to improving the efficiency of nitrogen fertilizer use. Previous studies have shown that the effectiveness of most AFN is less than 50% (Salimi et al., 2023), resulting in a large surplus of N in the soil. Compared to field cultivation, in protected cultivation, crops are densely planted and grown continuously, leading to higher and more frequent inorganic AFN inputs. Excess AFN can lead to increased greenhouse gas emissions, such as nitrous oxide(N_2_O), and may also cause soil acidification, salinization, and a decline in fertility in protected soils (Channab et al., 2023; Du et al., 2017; Shao et al., 2021). Our study attempts to ecologically reduce the SRFN using conventional field management practices that are easy to implement, such as irrigation or fertilization (**Figures 2**, **8**).

**Figure 8:**
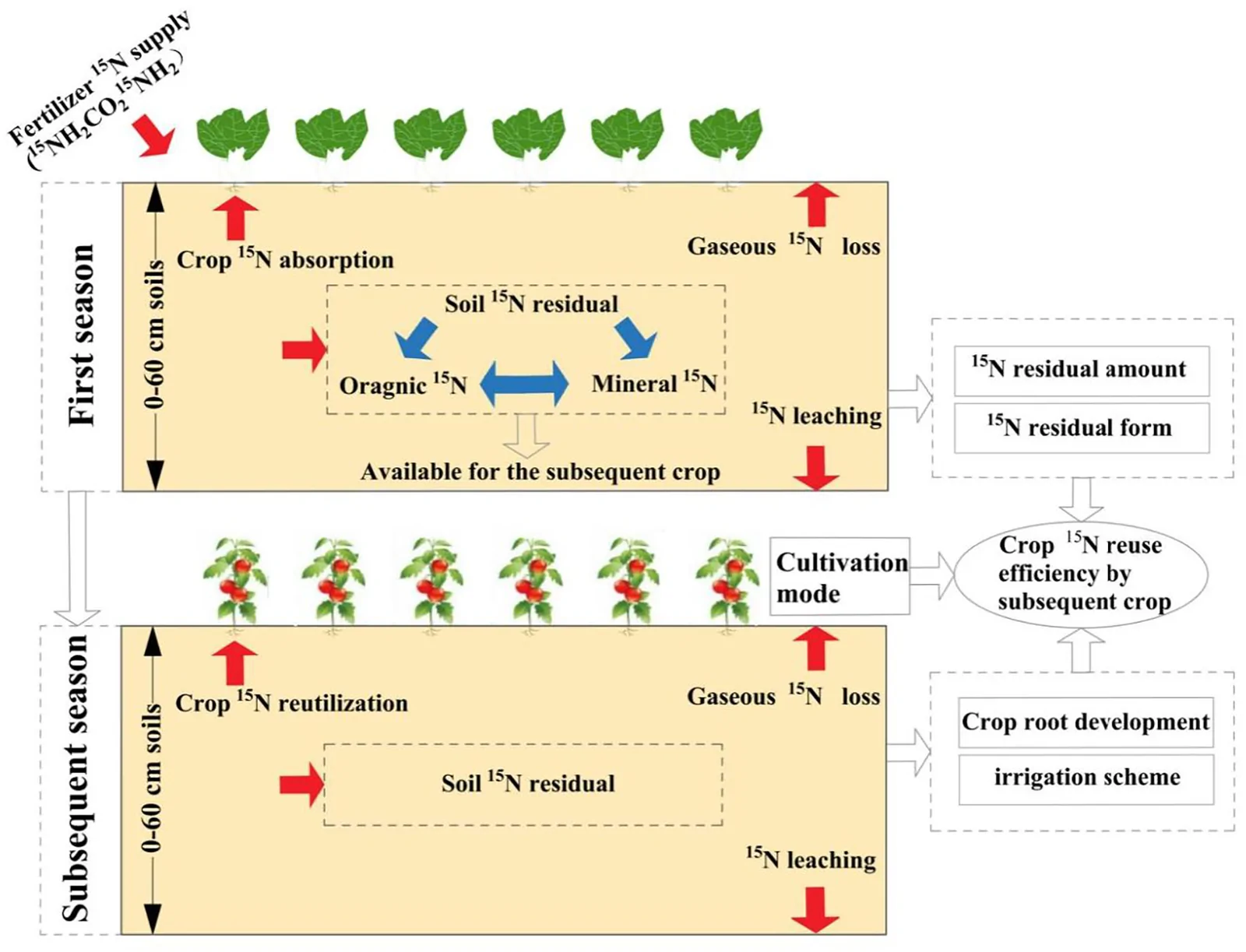
The fate and balance of previous soil ^15^N residue in the cultivation process of succeeding crops.

Pakchoi is a vital vegetable in many countries, essential for people’s daily nutrition. Its cultivation process has a high demand for N nutrients, requiring large amounts of inorganic AFN inputs. After cultivating pakchoi, the surplus N remaining in the soil has a long-term negative impact on the soil environment (Argento et al., 2024). In China, tomatoes are another widely grown vegetable crop that plays an important role in ensuring national food security (Yang et al., 2024). Previous research has shown that (Kim et al., 2025) crop rotation might be a feasible method to optimize field nutrient management. Therefore, this study used pakchoi as the plant material in the first crop season and tomato as a potential subsequent crop that might efficiently absorb the SRFN after pakchoi cultivation.

### 4.1 Soil moisture dynamics and SRFN distribution as affected by irrigation strategies

The soil moisture fluctuation at the 40–60 cm depth was more pronounced in the tomato-cultivated soil than in the pakchoi-cultivated soil. This phenomenon can primarily be attributed to differences in root distribution and water uptake depth between the two crops, as well as the disparity in growth period length and water consumption intensity. Tomato, as a deep-rooted crop, has a root system that can extend to the 40–60 cm layer or deeper, enabling it to directly absorb water from this depth and resulting in pronounced moisture fluctuations throughout its longer growth period (approximately 120 days). The strong transpiration during the fruiting stage further intensifies this fluctuation. In contrast, pakchoi is a shallow-rooted vegetable, with its root system mainly distributed in the 0–30 cm surface layer. With a growth period of only about 35 days, its shorter water consumption duration leaves insufficient time for significant changes in deep soil moisture before harvest.

The more developed root system of tomatoes (**Table 6**) might cause preferential flow through root pores (Bi et al., 2025; Cai et al., 2024), facilitating the movement of water carrying ^15^N. The more significant fluctuation in moisture content of the tomato soil in deeper layers (**Figure 3**) might support this explanation. Our study proved that the higher irrigation quota per irrigation event tended to promote the migration of ^15^N to deeper soil layers for both pakchoi and tomato cultivation soils (**Figure 4**), which might increase the risk of ^15^N loss (calculated by the balance, **Table 8**). This conclusion was consistent with Jin’s (Jin et al., 2022) result on the lettuce fields. Our results also suggested that, in practice, irrigation with high frequency but low quota is beneficial for maintaining moisture in the shallower soil layers, thereby reducing the leaching loss of SRFN. In our experiment, under high irrigation quotas, water migrate downward due to the surrounding barriers of the blocks, this might increase the leaching loss of ^15^N. In contrast, in actual production, high irrigation quotas will increase lateral water migration, moving N to the areas where crop roots cannot capture, and may thus increase the risk of AFN loss. It is worth noting that, since the value of the loss pathway was calculated using the difference method, this implies that we can only discuss the portion of ^15^N that remained unaccounted for in the mass balance, rather than fully elucidating the mechanisms underlying the loss.

### 4.2 Effects of previous N application rates on SRFN availability and crop uptake

The increase in AFN from N1 to N3 significantly affected the biomass accumulation of the subsequent pakchoi but has no significant impact on that of the subsequent tomato (**Table 6**). This might be because tomatoes require more N nutrients than pakchoi, and the SRFN amount under our experimental design was insufficient to promote the growth of the subsequent tomato noticeably. It was also possible that tomatoes did not effectively utilize urea-derived ^15^N. Urea hydrolysis increases soil alkalinity upon application, whereas tomatoes favor slightly acidic conditions; thus, tomatoes may rely more on native soil N than on residual urea N. This inference could be supported by the biomass and ^15^N uptake measurements (**Tables 6**, **7**): pakchoi accumulated a higher concentration of ^15^N per unit of biomass, while tomatoes absorbed a lower concentration of ^15^N despite producing substantially greater biomass. This inference might be supported by the measurement results of biomass and ^15^N absorption (**Tables 6**, **7**): pakchoi accumulated a higher concentration of ^15^N per unit of biomass, while tomatoes absorbed a lower concentration of ^15^N under a much greater amount of biomass. The recovery rate of SRFN by tomato was significantly higher than that by pakchoi, primarily due to differences in crop biological characteristics. Tomato has a long growth period (approximately 120 days) and a well-developed root system that can penetrate deeply into the 40–60 cm soil layer, enabling it to absorb mineral ^15^N leached into the deeper soil. In addition, its large biomass results in a substantially higher total N demand compared with pakchoi. In contrast, pakchoi has a short growth period of only about 35 days, with its root system mainly distributed in the shallow 0–30 cm layer, which limits both its absorption capacity and the duration of N uptake, resulting in a lower recovery rate of SRFN.

The ^15^N reuse amount was closely positively correlated with the SRFN content. Notable evidence was that under the TN3 treatment, the tomato ^15^N reuse amount was significantly higher than that under TN1 (**Table 7**). Correspondingly, the SRFN in the main root layer (0–40 cm) under TN3 was also significantly higher than under TN1 (**Figure 4**). However, there was no significant difference in the plant biomass between TN3 and TN1 (**Table 6**), suggesting that the amount of SRFN from the previous crop might have little impact on the biomass and total N accumulation in the subsequent tomato, but it would change the distribution ratio of the SRFN within the crop.

### 4.3 Responses of crop growth and SRFN uptake to irrigation strategies

In this experiment, W2 promoted plant biomass accumulation. This might be attributed to two reasons: (1) suitable moisture conditions helped to create a favorable rhizosphere environment, thus promoting crop root growth and development. An earlier study has shown that water management inevitably affected the rhizosphere soil moisture and the soil mineral nutrients, and in response, crop roots would make corresponding changes themselves to absorb the water and nutrients they need (Gruber et al., 2013). Crop roots could recognize the extremely microscopic water availability in their surrounding environment, leading to changes in root configuration, which ultimately are reflected in the root number, length, and weight (Bao et al., 2014). (2) Suitable moisture conditions helped to reduce the volatile N loss from the soil, such as the ammonia loss (Martin and Chapman, 1951) and promote the soil N mineralization (Jin et al., 2013; Xu et al., 2020), thereby creating more available N for crop absorption, which was the basic condition for the crop biomass accumulation. It is important to point out that the application of fresh N fertilizer during the subsequent crop growth period made it difficult to distinguish the relative contributions of SRFN and current-season applied N to crop production. Although the ^15^N labeling technique effectively traced the SRFN, the growth and yield formation of the subsequent crop were also dependent on the N supplied during the current season.

Under the same SRFN conditions, suitable soil moisture benefited the absorption of ^15^N by the subsequent crop (**Table 7**), which in turn improved the ^15^N reuse rate (**Figure 6**). This might be related to the enhanced crop root system under suitable moisture conditions (**Table 6**) and also related to the greater amount of soil mineral ^15^N triggered by such moisture conditions (**Figure 4**). Although our results and many previous studies suggested a positive correlation between crop ^15^N use rate and soil mineral ^15^N, (Liu et al., 2015) showed that crop ^15^N utilization was negatively correlated with soil mineral ^15^N since ^15^N and the other soil N were mineralized simultaneously, with ^15^N accounting for only a small portion of the total N pool. As a result, the mineralization amount of ^15^N was lower than that of the other N forms, which might reduce the opportunity for crop root systems to capture ^15^N and might lower the ^15^N absorption amount by plants.

### 4.4 ^15^N balance and its implications for water-N management in protected cultivation

From the perspective of ^15^N balance, in this study, over 70% of the SRFN from the first crop remained in the soil after the subsequent crop cultivation, and 4.4–12.6% was lost during the subsequent crop cultivation process (**Figure 8**). The loss proportion of ^15^N was lower than that observed in our earlier experiment of a tomato-tomato system (7.2–27.1%) and also lower than the results from Hu (Hu et al., 2001) in field studies, which indicated a total N loss of 25% (including 15% ammonia volatilization, 9% leaching, and 1% denitrification loss). This difference might be related to factors such as the crop types and the cultivation conditions. When the subsequent crop was pakchoi, a shallow-rooted crop, SRFN was located in the shallower soil layers and, thus, was less likely to be lost through leaching during the succeeding crop’s cultivation process. Furthermore, under protected cultivation with intensive and high-frequency cropping, SRFN was easier to absorb by the succeeding crop, making it less prone to loss compared to field cultivation.

Despite the implementation of optimized crop management practices, the recovery rate of residual nitrogen by subsequent crops remained relatively low (2.48%–8.95%), with over 70% of the residual nitrogen being immobilized in the soil organic pool. This indicates that in-season recovery is not the primary pathway for residual nitrogen utilization; rather, its immobilization in the soil organic pool and long-term mineralization release may hold greater agronomic and environmental significance. Therefore, the ecological effects of SRFN should not be evaluated solely based on the in-season crop recovery rate, but should be comprehensively assessed over longer temporal scales with respect to its contributions to soil nitrogen pool maintenance and emission reduction.

Our research demonstrated that the AFN rates in the previous crop or the irrigation strategy in the subsequent crop influences the balance or reuse of SRFN through various pathways. The AFN rates in the previous crop mainly affected the location, quantity, and form of SRFN. The irrigation strategy in the subsequent crop affected not only the location and form of ^15^N but also the root growth and development. Since the irrigation and fertilization measures inevitably affect the rhizosphere soil property, change the transformation of SRFN, and influence root growth and N absorption processes, in the future, more indicators should be observed, such as the soil indicators, including structure, permeability and microorganisms, the N form indicators, including microbial biomass N and lattice-fixed ammonia, as well as the crop indicators, including active root absorption area and plant N metabolism. These will help better understand the mechanisms of the efficient utilization of SRFN. One limitation of this study is that we did not determine the particle size distribution (soil texture) prior to the experiment. Soil texture may vary across different regions and can influence N uptake efficiency to some extent, which may potentially affect the absolute values. Another limitation is that the limited number of replications and the lack of observations for deep-layer ^15^N may also affect the applicability of the findings. Additionally, it should be noted that the experiment was conducted in a system of isolated plots separated by PVC boards on a small scale, which may have influenced water flow, root development, and the vertical movement of N, and thus may have influenced the applicability of the findings.

Through two independent ^15^N tracing experiments conducted under two rotation backgrounds, this study investigated the effects of previous nitrogen application rates (experiment in 2023) and subsequent irrigation strategies (experiment in2024) on the reuse and balance of soil residual fertilizer N. Our findings are consistent with the data presented and address the stated research objectives. This study contributes to understanding N behavior in soil and provides references for improving the utilization efficiency of applied N fertilizers in the investigated crops. Furthermore, by conducting two independent experiments, this study provides data support for understanding the residual effects of N under different fertilization levels or irrigation regimes.

## 5 Conclusions

Through two independent ^15^N tracing experiments under different rotation backgrounds, this study examined the effects of previous N application rates (2023) and subsequent irrigation strategies (2024) on SRFN reuse and balance. After subsequent crop cultivation, over 70% of SRFN remained in the soil, 4.4%–12.6% was lost, and 2.48%–8.95% of the applied fertilizer N was absorbed by the subsequent crop. In 2023, increasing the previous N rate from 80 to 160 kg N/ha significantly promoted biomass of subsequent pakchoi under pakchoi-pakchoi rotation, but had no significant effect on tomato biomass under pakchoi-tomato rotation. In 2024, the 70% limit for pakchoi and the 60%–70%–70% limit for tomato most significantly promoted root dry matter accumulation, increasing SRFN uptake by 0.45–1.01 kg/ha and 1.94–2.56 kg/ha, respectively, with the highest reuse rates of 4.05% and 8.95%. Correlation analysis indicated that SRFN reuse was influenced by its location, form, and quantity (2023), and by root biomass of the subsequent crop (2024). Overall, the pakchoi-tomato rotation with a 60%–70%–70% irrigation lower limit is recommended for ecological reduction of SRFN. This recommendation is derived from greenhouse-based experiments, and its extrapolation to open-field conditions may yield different outcomes. Therefore, further application of these findings warrants field validation under diverse environmental conditions. Moreover, the long-term agronomic and environmental effects of this residual nitrogen pool remain to be further elucidated through multi-year field experiments.

## Data Availability

The original contributions presented in the study are included in the article/supplementary material. Further inquiries can be directed to the corresponding authors.
