# Correction: Nitrogen uptake from soil residual fertilizer by subsequent crops: regulatory effects of previous nitrogen application rate and subsequent irrigation regime

**DOI:** 10.3389/fpls.2026.2009814

**Published:** 2026-10-07

**Authors:** Xiaohui Xu, Jingnan Chen, Yiting Cai, Ying Tian, Erzi Zhang, Maomao Hou, Fenglin Zhong, Junyang Lu, Jiajia Li

**Affiliations:** 1 College of Agricultural Science and Engineering, Hohai University, Nanjing, Jiangsu, China; 2 College of Horticulture and Landscape Architecture, Fujian Agricultural Vocational and Technical College, Fuzhou, Fujian, China; 3 College of Horticulture, Fujian Agriculture and Forestry University, Fuzhou, Fujian, China; 4 Jiangning District Water Affairs Bureau of Nanjing City, Nanjing, Jiangsu, China; 5 Plant Research and Development Laboratory, S’yong Cosmetics Manufacturing Co., Ltd., Changsha, Hunan, China

**Keywords:** facility agriculture, irrigation regime, nitrogen use efficiency, pakchoi, tomato

The funder “National Natural Science Foundation of China, (32402559)” was erroneously omitted.

“The author(s) declared that financial support was received for this work and/or its publication. This research was funded by the General Project of the Natural Science Foundation of Fujian Province (2023J01133579), the Fujian Modern Agricultural Vegetable Industry System Construction Project (2019-897), the Fujian Provincial Education Research Project for Middle-aged and Young Teachers (JAT231020), and the National Natural Science Foundation of China (32402559)”.

The original version of this article has been updated.

